# Molecular characterization of highly prevalent *Escherichia coli* and *Escherichia marmotae* resistant to extended-spectrum cephalosporins in European starlings (*Sturnus vulgaris*) in Tunisia

**DOI:** 10.1128/spectrum.02220-23

**Published:** 2023-09-29

**Authors:** Meriem Souguir, Pierre Châtre, Antoine Drapeau, Pauline François, Sana Azaiez, Sana Ncir, Jean-Yves Madec, Wejdene Mansour, Marisa Haenni

**Affiliations:** 1 Laboratoire de Recherche Biophysique Métabolique et Pharmacologie Appliquée, Faculté de Médecine Ibn Al Jazzar Sousse, Université de Sousse, Sousse, Tunisia; 2 Unité Antibiorésistance et Virulence Bactériennes, ANSES - Université de Lyon, Lyon, France; Taichung Veterans General Hospital, Taichung, Taiwan

**Keywords:** *E. marmotae*, starlings, Tunisia, ESBL, AmpC, plasmids, One Health, spread

## Abstract

European starlings are widespread migratory birds that have already been described as carrying bacteria resistant to extended-spectrum cephalosporins (ESC-R). These birds are well known in Tunisia because they spend the wintertime in this country and are hunted for human consumption. The goal of our study was to estimate the proportion of ESC-R in these birds and to characterize the collected isolates using whole-genome sequencing. Results showed that 21.5% (42/200) of the birds carried either an extended-spectrum beta-lactamase (ESBL) or an acquired AmpC gene. Diverse *bla*_CTX-M_ genes were responsible for the ESBL phenotype, *bla*_CTX-M-14_ being the most prevalent, while only *bla*_CMY-2_ and one *bla*_CMY-62_ were found in AmpC-positive isolates. Likewise, different genetic determinants carried these resistance genes, including IncHI2, and IncF plasmids for *bla*_CTX-M_ genes and IncI1 plasmids for *bla*_CMY-2_ genes. Three chromosomally encoded *bla*_CTX-M-15_ genes were also identified. Surprisingly, species identification revealed a large proportion (32.7%) of *Escherichia marmotae* isolates. This species is phenotypically indistinguishable from *Escherichia coli* and has obviously the same capacity to acquire ESC-R genes. Our data also strongly suggest that at least the IncHI2/pST3 plasmid can spread equally between *E. coli* and *E. marmotae*. Given the potential transmission routes between humans and animals, either by direct contact with dejections or through meat preparation, it is important to closely monitor antimicrobial resistance in European starlings in Tunisia and to set up further studies to identify the sources of contamination of these birds.

**IMPORTANCE:**

The One Health concept highlighted knowledge gaps in the understanding of the transmission routes of resistant bacteria. A major interest was shown in wild migratory birds since they might spread resistant bacteria over long distances. Our study brings further evidence that wild birds, even though they are not directly submitted to antibiotic treatments, can be heavily contaminated by resistant bacteria. Our results identified numerous combinations of resistance genes, genetic supports, and bacterial clones that can spread vertically or horizontally and maintain a high level of resistance in the bird population. Some of these determinants are widespread in humans or animals (IncHI2/pST3 plasmids and pandemic clones), while some others are less frequent (atypical IncI1 plasmid and minor clones). Consequently, it is essential to be aware of the risks of transmission and to take all necessary measures to prevent the proportions of resistant isolates from increasing uncontrollably.

## INTRODUCTION

Antimicrobial resistance (AMR) is a complex phenomenon that knows no barriers and is threatening humans, animals, and the environment worldwide. Enterobacterales resistant to extended-spectrum cephalosporins (ESC-R) and carbapenems (CP) are among the most significant issues. AMR has long been overlooked in wildlife (1) but, for the last decade, clinically relevant AMR bacteria—including ESC-R and CP-R—have been isolated from various wild bird species on all continents, including vultures, gulls, buzzards, or red kites (2 – 4). The presence of resistance genes and antibiotic-resistant bacteria in wildlife is most probably an indicator of anthropogenic contamination rather than resistance selection since wild animals are usually not directly exposed to antibiotics (5). Consequently, wild birds can be both reservoirs and spreaders of resistance genes or bacteria of human origin. Moreover, migratory birds, through their movements over long distances, can be partly responsible for the worldwide transmission of resistance genes (6).

Starlings (*Sturnus vulgaris* or European starling) are wild birds native to Europe and South-East Asia that have later reached to North America (7), Australia, New Zealand, and South Africa (8). They are great travelers, migrating over long distances (1,000–1,500 km) searching for food in winter (8). Starlings are so widespread and problematic that they were classified among the three worst invasive birds on the World Conservation Union List (9, 10). They can be potential reservoirs of bacteria as they have been recurrently described as carrying *Campylobacter jejuni* isolates (11, 12). Cefotaxime- and ciprofloxacin-resistant *Escherichia coli* were also reported from European starlings in Canada, and one ESC-R SHV-12-producing *E. coli* was reported from a Spotless starling in Spain (13 – 15).

Tunisia is on the migratory route of starlings that are traveling from Europe for the winter season before leaving in spring. During this 6-month period, starlings are traditionally hunted for human consumption and sold alive on local markets. While consumption of meat produced in intensive farms (such as poultry meat) where animals are treated with antibiotics has been widely studied, the risk of consuming wild animal meat has been much less evaluated. Our study thus aimed at isolating and characterizing ESC-R and CP-R Enterobacterales, in order to determine whether these birds carry resistant bacteria, which could be further transmitted through the food chain or by fecal contamination of domestic animals and the environment.

## RESULTS

### Prevalence and genetic diversity of ESBL/AmpC-producing isolates

Over the 200 samples tested, corresponding to 200 birds, 42 (42/200, 21.5%) presented growth on plates containing cefotaxime, while none of them grew on imipenem-containing plates. One isolate was picked per plate/bird, except for one plate that presented two colony morphologies, leading to a final collection of 43 Enterobacterales.

**Fig 1:**
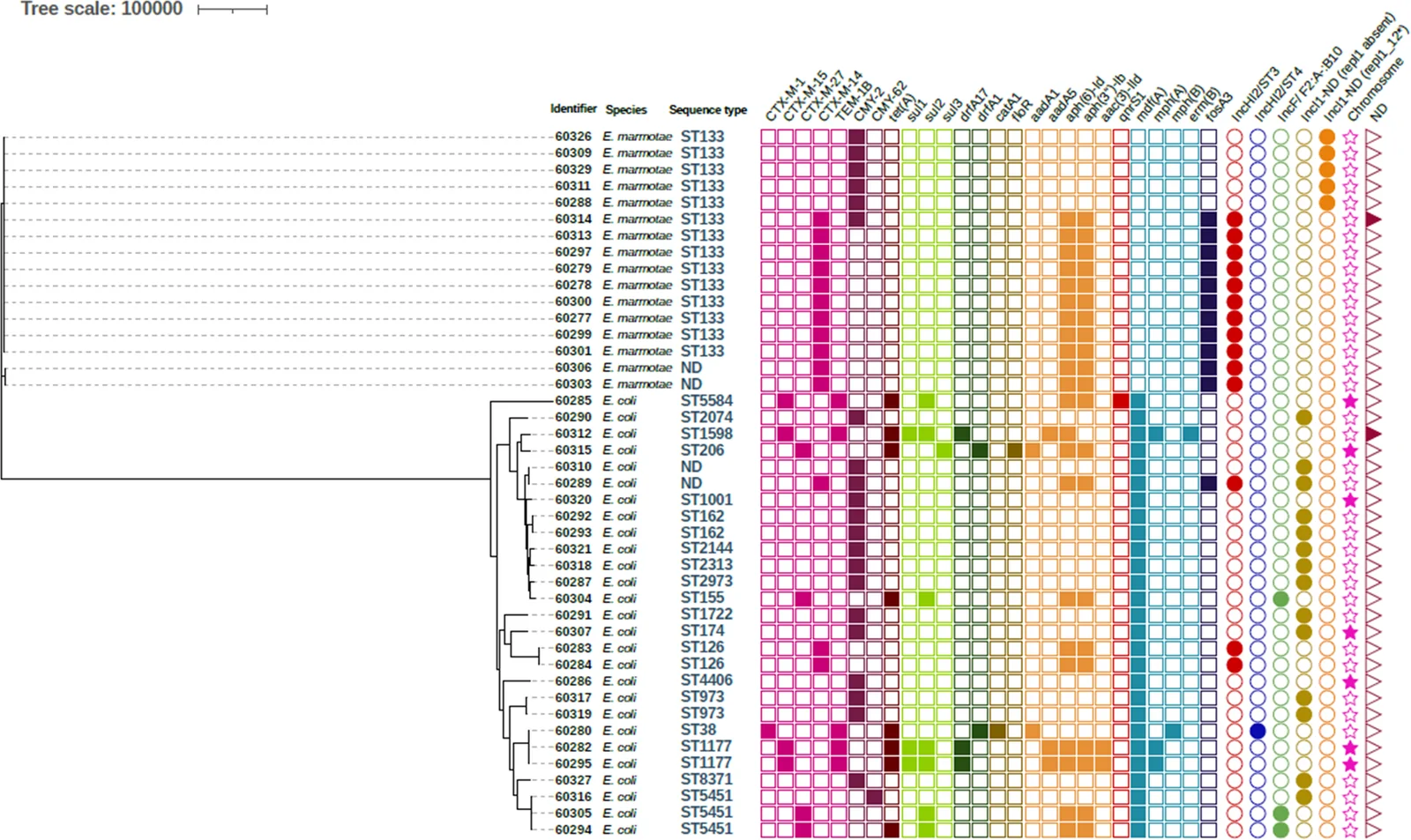
SNP phylogeny of the *E. coli* and *E. marmotae* isolates using iTOL. Two isolates (#60314 and #60289) presented both the *bla*_CTX-M-14_ and the *bla*_CMY-2_ gene. The *bla*_CTX-M-14_ gene was located on an IncHI2/pST3 plasmid in both isolates, while the *bla*_CMY-2_ gene was located on an IncI1 plasmid in isolate #60289 and on an undetermined genetic element in isolate #60314.

Isolates were identified as *E. coli* (*n* = 27, 62.8%) and *Escherichia marmotae* (*n* = 16, 37.2%). All *E. marmotae* isolates belonged to ST133, except for two presenting a common unknown sequence type (ST) (Table S1; Fig. 1). ST133 isolates were genetically linked and clustered according to their geographical origin; isolates from Gabès were nearly identical with 0–4 single-nucleotide polymorphism (SNP) differences, but differed by 42 SNPs from the Bizerte isolate and by 192–198 SNPs from the Zaghouan isolates (Table S2; Fig. S1). Only one isolate from Gabès (#60301) was genetically closer to the Bizerte isolate (#60314) than from the other isolates from Gabès. On the contrary, *E. coli* isolates belonged to 20 different sequence types, including 1 unknown and 19 already described STs: ST5451 was found in three different samples, while ST126, ST162, ST973, and ST1177 were each found in two samples. Three STs belonged to pandemic clones, namely ST38, ST155, and ST162.

### ESBL and AmpC resistance genes

Twenty-three isolates (12 *E. coli* and 11 *E. marmotae*; 23/200, 11.5%) displayed an extended-spectrum beta-lactamase (ESBL) phenotype, and 22 (16 *E. coli* and 6 *E. marmotae*; 11%) an AmpC phenotype, among which two isolates presented a mixed ESBL/AmpC phenotype (Table S1). The ESBL phenotype was due to the presence of the *bla*_CTX-M-14_ gene in all *E. marmotae* isolates, while it was conferred by the *bla*_CTX-M-15_ (*n* = 4), *bla*_CTX-M-27_ (*n* = 4), *bla*_CTX-M-14_ (*n* = 3), and *bla*_CTX-M-1_ (*n* = 1) genes in *E. coli* isolates (Table S1). AmpC-positive isolates systematically displayed the *bla*_CMY-2_ gene, except for one *E. coli* isolate that presented the *bla*_CMY-62_ gene. One *E. coli* and one *E. marmotae* co-harbored the *bla*_CTX-M-14_ and *bla*_CMY-2_ genes. The 14 *bla*_CTX-M-14_-positive isolates originated from the three cities where birds were caught (Gabès, Bizerte, and Zaghouan), while 20/21 of the *bla*_CMY-2_-positive isolates originated from Zaghouan.

### Antibiotic susceptibility testing and associated resistance genes

All ESBL-producing isolates presented resistance to non-β-lactam antibiotics, while those only displaying the *bla*_CMY-2_ gene were susceptible to all non-β-lactam antibiotics tested (Table S1). *E. marmotae* isolates presented globally less resistance to non-β-lactams than *E. coli*. Streptomycin was the most frequently found resistance in both *E. coli* and *E. marmotae* isolates (51.2%), followed by tetracyclines, sulfonamides, and trimethoprim (25.6%, 23.3%, and 11.6%, respectively). Resistance proportions were less than 5% to enrofloxacin (2.3%) and gentamicin (4.7%) (Table 1). Aminoglycoside resistance was principally due to the co-occurrence of the *aph(6)-Id* and *aph(3″)-Ib* genes (*n* = 22), in both *E. coli* (*n* = 11) and *E. marmotae* (*n* = 11) (Table S1). The *fosA3* gene conferring high-level resistance to fosfomycin was also found in both *E. coli* (*n* = 3) and *E. marmotae* (*n* = 11). On the contrary, genes conferring resistance to sulfonamides [*sul1* (*n* = 3), *sul2* (*n* = 7), and *sul3* (*n* = 1)], trimethoprim [*drfA1* (*n* = 2) and *drfA17* (*n* = 3)], tetracyclines [*tet*(A), *n* = 9] as well as quinolones [*qnrS1* (*n* = 1)] were only identified in *E. coli*. Finally, the *mcr-1* gene present in the ST38 *E. coli* isolate was truncated by the IS*Apl1*, explaining why colistin resistance was not observed.

**TABLE 1:** Antimicrobial susceptibility phenotypes of all 43 *E. coli* and *E. marmotae* isolates characterized in this study^*a*^

	*Escherichia coli* (*n* = 27)		*Escherichia marmotae* (*n* = 16)		Total (*n* = 43)	
	*n*	%	*n*	%	*n*	%
Amoxicillin	27	100.0	16	100.0	43	100.0
Amoxicillin + clavulanic acid	21	77.8	6	37.5	27	62.8
Ceftiofur	27	100.0	16	100.0	43	100.0
Cefquinome	12	44.4	13	81.25	25	58.1
Ceftazidime	24	88.9	6	37.5	30	69.8
Cefoxitin	17	63.0	5	31.25	22	51.2
Ertapenem	0	0.0	0	0.0	0	0.0
Sulfonamides	9	33.3	1	6.25	10	23.3
Trimethoprim	5	18.5	0	0.0	5	11.6
Nalidixic acid	7	25.9	0	0.0	7	16.3
Enrofloxacin	1	3.7	0	0.0	1	2.3
Colistin	0	0.0	0	0.0	0	0.0
Chloramphenicol	4	14.8	0	0.0	4	9.3
Florfenicol	2	7.4	0	0.0	2	4.7
Tetracycline	10	37.0	1	6.25	11	25.6
Gentamicin	2	7.4	0	0.0	2	4.7
Streptomycin	12	44.4	10	62.5	22	51.2
Kanamycin	0	0.0	0	0.0	0	0.0
Tobramycin	2	7.4	0	0.0	2	4.7
Netilmicin	0	0.0	0	0.0	0	0.0
Apramycin	0	0.0	0	0.0	0	0.0
Amikacin	0	0.0	0	0.0	0	0.0

^*a*^ Tests were performed using disc diffusion, except for colistin, for which the minimum inhibitory concentration was determined using broth microdilution.

### Characterization of the genetic determinants carrying ESBL/AmpC genes

Combined analysis of Southern blots, short-read, and long-read sequences proved that the plasmid-borne *bla*_CTX-M_ genes were carried by large IncHI2 or IncF plasmids. The *bla*_CTX-M-15_ gene was carried on the chromosome in three isolates, and on an undetermined genetic determinant in the fourth one. All four *bla*_CTX-M-15_ genes were preceded by the IS*Ecp1* element and the two chromosomally encoded genes were followed by a tryptophan synthase beta chain gene in a genetic environment similar to the one described by Guenther et al. in the ST38 IMT37356 isolate (16). Alternatively, *bla*_CMY-2_ genes were carried either on IncI1 plasmids or on the chromosome.

#### *bla*_CTX-M-14_-carrying plasmids

**Fig 2:**
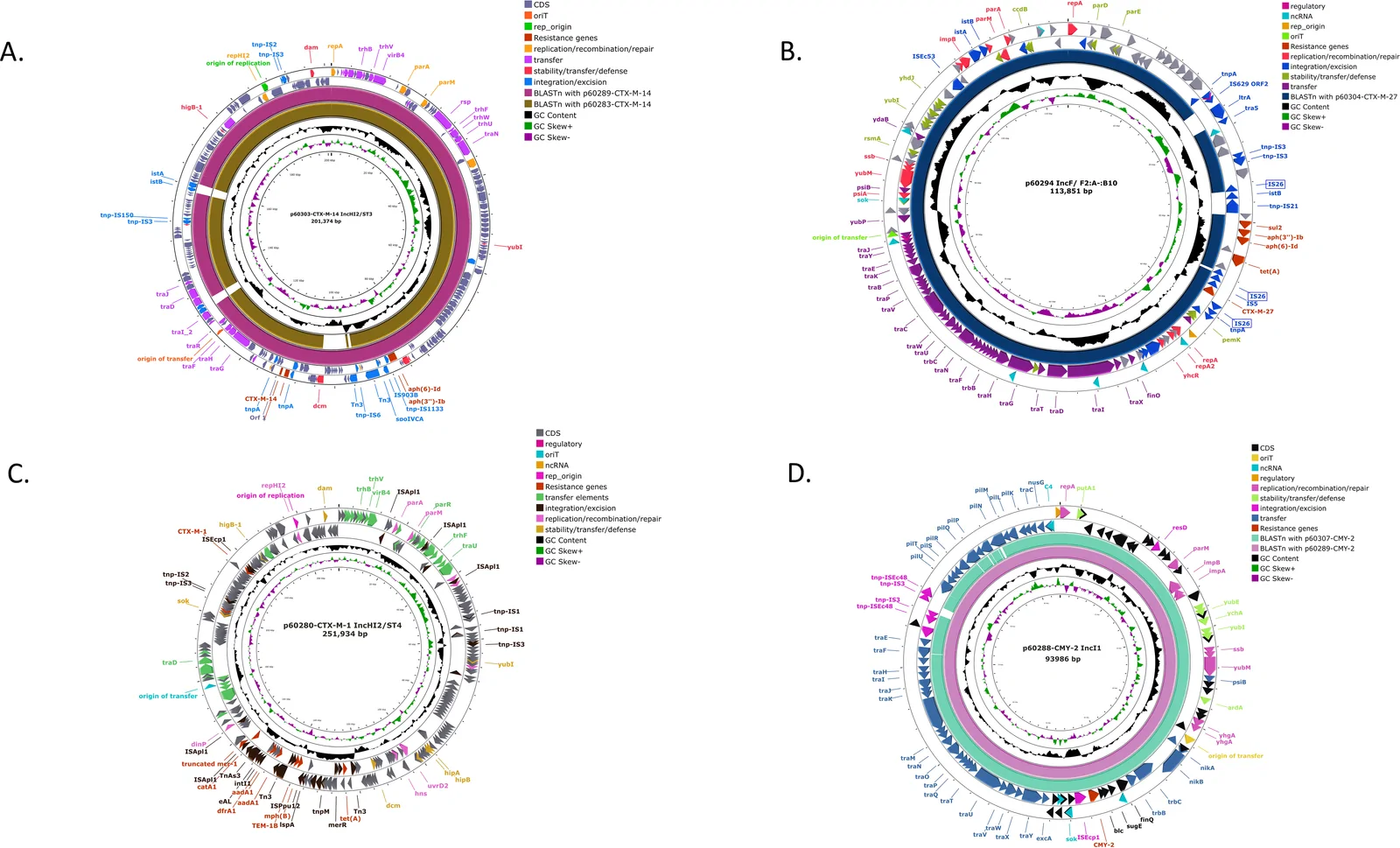
Visualization of the long-read sequenced plasmids carrying the *bla*_CTX-M-14_ (A), *bla*_CTX-M-27_ (B), *bla*_CTX-M-1_ (C), and the *bla*_CMY-2_ (D) genes using the PROKSEE tool.

**Fig 3:**
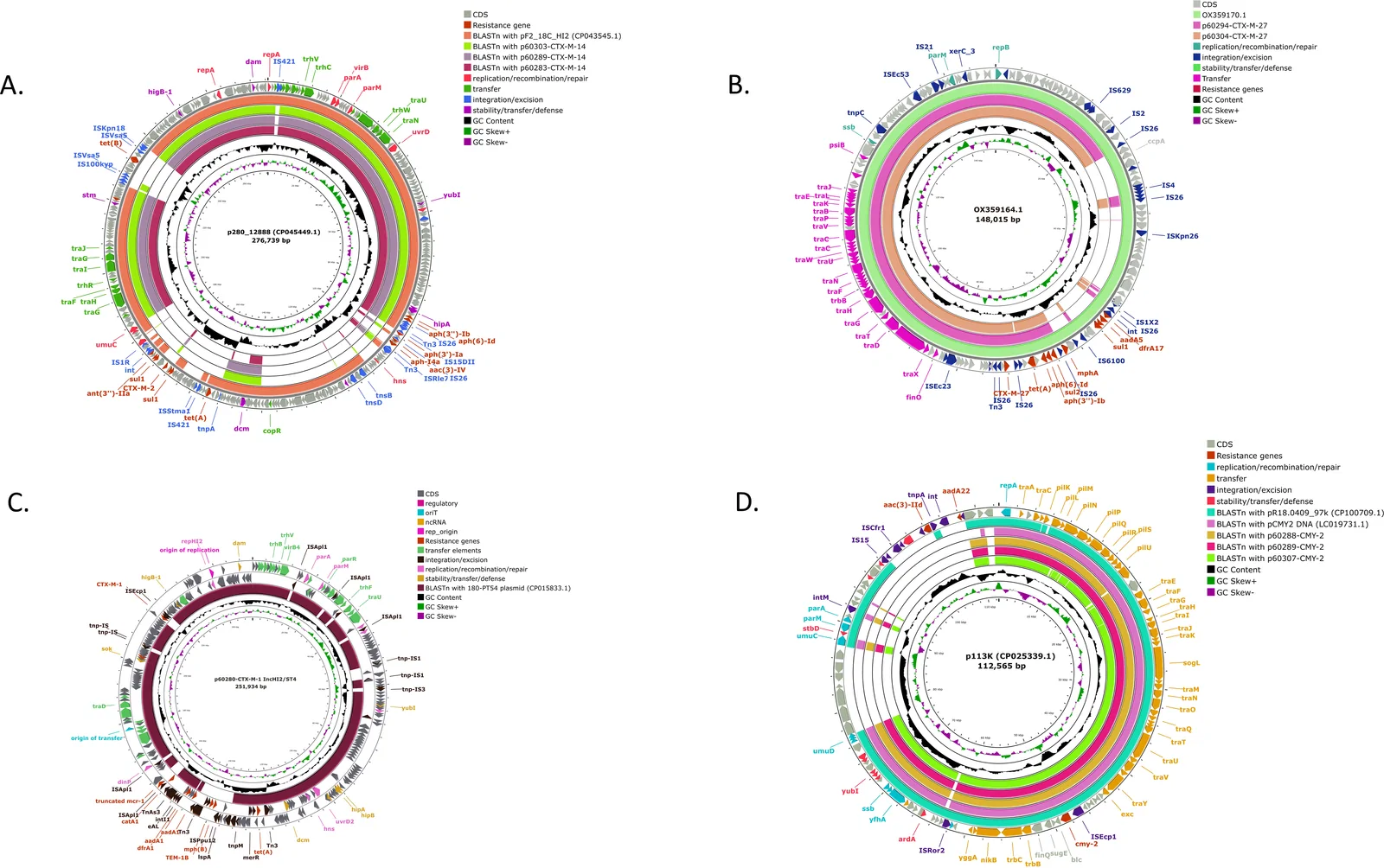
Comparison of plasmids carrying the *bla*_CTX-M-14_ (A), *bla*_CTX-M-27_ (B), *bla*_CTX-M-1_ (C), and the *bla*_CMY-2_ (D) genes with the closest plasmids found in the publically available databases using the PROKSEE tool.

The *bla*_CTX-M-14_ gene, which was found both in *E. coli* (*n* = 3, including two clonal ST126 isolates with no SNP difference) and *E. marmotae* (*n* = 11) isolates, was systematically carried by IncHI2/ST3 plasmids and co-harbored the *fosA3*, *aph(6)-Id*, and *aph(3″)-Ib* genes. Three plasmids were fully sequenced, namely p60283-CTX-M-14 (190,524 bp), p60289-CTX-M-14 (199,906 bp), and p60303-CTX-M-14 (201,374 bp). They were nearly identical with a coverage >97% and presented a GC content of 45.3% (Fig. 2A). The *bla*_CTX-M-14_ and *fosA3* genes were identified in the IS*26*-ΔIS*Ecp1-bla*_CTX-M-14_-ΔIS*903B-fosA3*-orf1-Δorf2-IS*26* genetic context, which is identical to the type V *fosA3* surrounding region also identified on an IncHI2/pST3 plasmid by Yang et al. (17). Downstream were the *aph(6)-Id* and *aph(3″)-Ib* genes (also named *strA-strB*), preceded by an IS*903B*-ΔIS*1133* element identical to the one described by Jarocki et al., which was identified on an *E. coli* IncHI2/pST3 plasmid isolated from a piglet in Australia (pF2_18C_HI2; accession number: CP043545.1) (18). The three *bla*_CTX-M-14_-carrying plasmids exhibited 99% identity and a 94%–97% coverage with this pF2_18C_HI2 plasmid and with p280_128 (accession number: CP045449.1; *Salmonella enterica* subsp. serovar Schwarzengrund isolates collected from a poultry in Brazil) (19). Both plasmids lacked the *bla*_CTX-M-14_ and *fosA3* genes, but p280_12888 displayed a *bla*_CTX-M-2_ gene (Fig. 3A).

#### *bla*_CTX-M-27_-carrying plasmids

The *bla*_CTX-M-27_ gene was found in four *E. coli* isolates, including the two clonal ST5451 ones (0 SNP difference). It was carried on IncF/F2:A-:B10 plasmids in three isolates and on the chromosome in the fourth one. In this latter isolate, the *bla*_CTX-M-27_ gene was preceded by an IS*908B* element and the isolate additionally presented the *aadA1*, *aph(6)-Id*, *aph(3″)-Ib*, *tet*(A), *sul3, floR,* and *dfrA1* resistance genes. The two fully sequenced p60294-CTX-M-27 (113,851 bp) and p60304-CTX-M-27 (109,448 bp) were highly similar (>99% identity, GC content around 51.9%) and co-harbored the *aph(6)-Id*, *aph(3″)-Ib*, *tet*(A), and *sul2* resistance genes (Fig. 2B). In both plasmids, the *bla*_CTX-M-27_ gene was flanked by two copies of the IS*26*, while the downstream IS*26* and a third IS*26* surrounded the *sul2-aph(3″)-Ib-aph(6)-Id-tet*(A) genes. Comparisons with the NCBI database showed that both plasmids shared >95% identity and 99.9% coverage with two plasmids (OX359170.1 and OX359164.1) found in *E. coli* strains isolated from human urine samples in a hospital in Norway (Fig. 3B). These two plasmids contained the same resistance genes found in the plasmids of the present study as well as an additional *sul2* gene and shared the same genetic organization with flanking IS*26* elements.

#### *bla*_CTX-M-1_-carrying plasmid

The *bla*_CTX-M-1_ gene was carried by a large (251,934 bp) IncHI2/ST4 plasmid named p60280-CTX-M-1 which co-harbored the *bla*_TEM-1B_, *mph*(B), two copies of *aadA1*, *dfrA1*, *catA1*, *tet*(A), and a truncated *mcr-1* gene (Fig. 2C). The *bla*_CTX-M-1_ gene was preceded by an IS*Ecp1* insertion sequence, but not located close to other resistance genes. p60280-CTX-M-1 shared 99.9% sequence identity and 87% coverage with the 240,662 bp 180-PT54 plasmid (CP015833.1) which was found in an *E. coli* O157 strain isolated from a human fecal sample with diarrhea in 2012 in the United Kingdom (20). The genetic backbones of these two plasmids were similar but they differed in the resistance region, since only the *dfrA1* and *tet*(A) genes were shared (Fig. 3C).

#### *bla*_CMY-2_-carrying plasmids

The 21 *bla*_CMY-2_ genes and the unique *bla*_CMY-62_ gene were identified in six *E. marmotae* ST133 and 16 *E. coli* belonging to 15 different STs (two isolates belonged to ST162). The *bla*_CMY-62_-positive ST5451 differed by 17 SNPs from the two *bla*_CTX-M-27_-positive ST5451 isolates, while the *E. marmotae* ST133 were identical (0–5 SNPs) when originating from the same city and differed by nearly 200 SNPs when originating from Zaghouan or Bizerte (Table S2). Isolates were all devoid of additional resistance genes, except 16 isolates which carried the chromosomally encoded *mdf*(A) gene, and two (one *E. coli* and one *E. marmotae*) which carried the *aph(6)-Id*, *aph(3″)-Ib,* and *fosA3* genes, together with the *bla*_CTX-M-14_ gene, on an additional IncHI2 plasmid. Seventeen *bla*_CMY-2_ genes and the *bla*_CMY-62_ gene were carried on ca. 90 kbp IncI1 plasmids, three *bla*_CMY-2_ genes were chromosomally encoded (#60286, #60307, and #60320), while the genetic determinant of the last *bla*_CMY-2_ gene could not be determined (Table S1; Fig. 1). All IncI1 plasmids from *E. coli* were untypable since they lacked the *repI1* gene, while those from *E. marmotae* presented an unknown repI1 allele close to repI1_12. The four other pMLST alleles were identical in all *E. marmotae* and *E. coli* isolates and belonged to the 29/15/11/3 allelic profile.

Three IncI1 plasmids were fully sequenced, which showed 99% identity over their total length and a GC content of 49.4% (Fig. 2D). The AmpC gene was found on an IS*Ecp1-bla*_CMY-2-_ *blc-sugE* fragment (3,735 bp) integrated in the *finQ* gene, a fragment also observed in Illumina sequences from other *bla*_CMY-2_-positive isolates. These plasmids shared 99% identity over up to 90% of their length with p113k (CP025339.1), pR18.0409_97k (CP100709.1), and pCMY2_DNA (LC019731.1). p113k and pR18.0409_97k were both isolated from *Salmonella enterica* serovar Typhimurium, the first one from a sick pig (*Sus scrofa domesticus*) (21) and the second one from a human stool sample, while pCMY2_DNA was found in an *E. coli* from human stool (22) (Fig. 3D). These three plasmids carried a *bla*_CMY-2_ gene, presented a pMLST closely related to our starling isolates (1/4/15/11/3 assigned to pST217 for p113k; 2/3/15/11/3 for pCMY2_DNA; and 1/4/15/11/2 assigned to pST52 for pR18.0409_97k) and all originated from Taiwan.

## DISCUSSION

ESC-R *E. coli* have spread in all One Health sectors including wildlife (23), so that they have become a key indicator in the global monitoring of AMR and a marker of environmental contamination related to human activity (24 – 26). ESBL-producing *E. coli* isolates were first described from wild birds in 2006 in Portugal (27) and numerous cases from a wide variety of bird species have been reported since then throughout the world (28). In this study, we observed a high prevalence of ESC-R *Escherichia* spp. isolates (21.5%) in European starlings, due to the presence of both ESBL-conferring (11.5%) and AmpC-conferring (11.0%) resistance genes. However, the origin of these resistance genes and resistant bacteria remains unknown; indeed, they might have been acquired in Tunisia as well as on the birds’ migratory routes. This is the first description of ESC-R-positive European starlings in North Africa, after their report in North America where 4% of the birds tested were ESC-resistant (13), and the detection of one SHV-12-positive *E. coli* in a Spotless starling in Spain (15). The carriage of ESC-R determinants in these widespread migratory birds is of concern and further studies are needed to decipher the routes of contamination, including through meat consumption, and the risk of contamination for humans and animals in contact. Also, other factors that play an important role in the evolution of the bird microbiota, such as diet, sex, age, feeding habits, and geographical location—which were not recorded here and are often overlooked—should be taken into account in future studies.

Here, 62.8% of the isolates were identified as *E. coli* (*n* = 27). These isolates were genetically highly diverse since they belonged to 20 different STs, indicating the absence of the large-scale spread of one or two successful clones among these birds that live in large groups. Only ST38, ST155, and ST162 belonged to pandemic clones. The remaining 37.2% (n=16) were identified as *E. marmotae,* among which 14/16 belonged to ST133. These isolates mainly clustered according to their city of origin, differing by 0–5 SNPs when originating from the same city and by 45 or 198 SNPs when originating from different cities (Table S2). This strongly suggests inter-individual transmissions and micro-evolutions at the city level. Only one *E. marmotae* from Gabès (#60313) clustered with the unique isolate collected from Bizerte (#60314); this genetic proximity cannot be explained since the two cities are 475 km apart. *E. marmotae*, renamed in 2015 after its host *Marmota himalayana* (29)*,* was formerly known as *E.coli* cryptic clade V, a clade especially associated with birds (30). This new species, which is phenotypically identical to *E. coli*, has since then been reported as a *bla*_CTX-M-1_ carrier from an Alpine marmot in a Belgian zoo (31), and as a *bla*_CTX-M-32_ carrier from a red deer in Poland (32); these reports, in addition to the starlings’ samples here, suggest that wild animals are preferential hosts of *E. marmotae*. A recent publication analyzing the genomes of 41 *E. marmotae* isolates suggested that this species can be pathogenic for humans but is still carrying few resistance determinants, even if sporadic cases of ESBL- and carbapenemase-producing isolates were reported (33). Our study revealed that *E. marmotae* can be a frequent carrier of AmpC- and ESBL-conferring genes that can be shared with *E. coli* isolates present in the same niche.

The ESBL phenotype was due to the presence of four different genes (*bla*_CTX-M-1_, *bla*_CTX-M-15_, *bla*_CTX-M-14_, and *bla*_CTX-M-27_) but only *bla*_CTX-M-14_ was identified in *E. marmotae*. The *bla*_CTX-M-14_ gene was carried by an IncHI2/ST3 plasmid in all *E. coli* and *E. marmotae* isolates, suggesting that this plasmid can spread easily and equally in both *Escherichia* species. This plasmid was also geographically widespread since it has been isolated in all three cities where birds were captured, even though it was much less present in Zaghouan. The backbone of this IncHI2 plasmid has already been described in *E. coli* and *Salmonella* Schwarzengrund isolates displaying different resistance genes. The resistance gene modules *aph(6)-Id-aph(3″)-Ib* and *bla*_CTX-M-14_-*fos3* described here were each flanked by insertion sequences and displayed genetic organizations already identified in other non-identical IncHI2/pST3 plasmids, indicating the plasticity of the resistance region. The presence of the *fosA3* gene is of concern since fosfomycin is one of the last resort antibiotics to treat carbapenemase-producing Gram-negative bacteria. The *fosA3* gene has often been associated with ESBL-conferring genes (34), including on IncHI2 plasmids in the same genetic context as described here in both *E. coli* and *E. marmotae* (17, 35), but it has only been reported twice in wild birds: in a German black kite and a frigate bird in a pristine Brazilian atoll (36, 37). Its presence in migratory birds living in large colonies favoring close contacts and genetic transfers should thus be monitored.

The second genetic determinants shared between *E. coli* and *E. marmotae* were the IncI1/*bla*_CMY-2_ plasmids, which have only been identified in Zaghouan. These plasmids only carried the *bla*_CMY-2_ gene with no additional gene conferring resistance to non-β-lactam antibiotics, as often seen in IncI1/pST2 and pST12 subtypes (38). The IS*Ecp1-bla*_CMY-2-_ *blc-sugE* fragment, which is a common genetic environment for *bla*_CMY-2_, was always found in the *finQ* gene (38, 39). All plasmids were genetically highly similar, except that those identified in *E. coli* lacked the *repI1* allele. Given the rare allelic profile identified in both *E. coli* and *E. marmotae* for the four other genes of the pMLST scheme, we can hypothesize that the IncI1 plasmid with the complete pMLST profile is the common ancestor, which then underwent recombination and excision before spreading in *E. coli*. Interestingly, plasmids showing the closest pMLST types all originated from humans or pigs in Taiwan, except two originating from a dog in the UK and from an unknown origin in Sweden.

In conclusion, our study revealed a surprisingly high prevalence of ESC-R isolates in European starlings in Tunisia. This was mostly due to the epidemic success of the *bla*_CTX-M-14_/IncHI2/pST3 mostly in Bizerte and Gabès, and *bla*_CMY-2_/IncI1 plasmids exclusively in Zaghouan. In addition, a few more sporadic resistance determinants were found, such as *bla*_CTX-M-27_/IncF/F2:A-:B10, *bla*_CTX-M-1_/IncHI2/ST4, and the chromosomally encoded *bla*_CTX-M-15_ genes. Our results also highlighted the importance of *E. marmotae—*and notably the ST133 lineage—as an ESC-R commensal bacterial species in wild birds, in coherence with other reports of *E. marmotae* in wildlife. The absence of data on the resistance genes/plasmids found in humans, animals, and the environment of these three cities, and especially in the olive farms that are seasonally invaded by starlings, is a limitation of this study—as well as a potential perspective for further studies, since the real risk of AMR transmission cannot be assessed.

## MATERIALS AND METHODS

### Wild bird sampling and bacterial isolation

Wild birds (*n* = 200) were purchased alive on the Sousse market, Tunisia, between January and February 2022 during the legal hunting period. Birds were caught in Bizerte, Zaghouan, or Gabès (Fig. S1), and kept in aviaries for as short a time as possible, without being fed. The intestine of each bird was collected immediately after the death of the bird using a sterile scalpel and stored at −20°C. Intestinal content was emptied in 10 mL of Trypto-casein soy broth (Biokar), homogenized and incubated for 18–24 h at 37°C. Overnight cultures were inoculated on selective MacConkey agar plates supplemented with cefotaxime or imipenem (2 mg/L), for the detection of ESC- or CP-resistant Enterobacterales. One colony per morphology and per plate was picked up. Identification was performed using API20E galleries (bioMérieux).

### Antimicrobial susceptibility testing

Susceptibility testing was performed on all 43 non-duplicate *E. coli* and *E. marmotae* using the disc diffusion method on Mueller-Hinton agar, according to the guidelines and clinical breakpoints of the Antibiogram Committee of the French Society for Microbiology (CA-SFM; www.sfm-microbiologie.org). The *E. coli* ATCC 25922 strain was used as quality control. A total of 16 β-lactam (amoxicillin, piperacillin, ticarcillin, amoxicillin/clavulanic acid, piperacillin/tazobactam, ticarcillin/clavulanic acid, cefalotin, cefuroxime, cefotaxime, ceftiofur, ceftazidime, cefoxitin, cefepime, cefquinome, aztreonam, and ertapenem) and 14 non-β-lactam (tetracycline, kanamycin, tobramycin, gentamicin, amikacin, apramycin, netilmicin, streptomycin, florfenicol, chloramphenicol, sulfonamides, trimethoprim, nalidixic acid, and enrofloxacin) antibiotics was tested. ESBL producing Enterobacterales were detected using the Double Disc Synergy Test. Minimum inhibitory concentrations were determined by broth microdilution for colistin, according to the European Committee for Antimicrobial Susceptibility Testing.

### Illumina short-read sequencing and data analyses

DNA was extracted using the NucleoSpin Microbial DNA extraction kit (Macherey-Nagel, Hoerdt, France) and sequencing was performed on a NovaSeq 6000 instrument (Illumina, San Diego, CA, USA). Quality control of the reads was performed using FastQC and low-quality sequences were trimmed using Trimmomatic v0.39. *De novo* assembly was performed using Shovill v1.0.4 and the quality of assemblies was assessed using QUAST v5.0.2 (Table S3). Identification was performed using Kraken (https://github.com/DerrickWood/kraken), STs according to Achtman’s MLST scheme, resistance genes and virulence factors were determined using the CGE online tools (http://www.genomicepidemiology.org/) MLSTFinder v2.0, ResFinder v4.1, and VirulenceFinder 2.0.3, while replicon content and plasmid formula were identified using PlasmidFinder 2.0.1 and pMLST 2.0. Serotypes were determined using SeroTypeFinder2.0. Detection of the phylogenetic groups of each *E. coli* isolate was performed *in silico* using Clermontyping online tool (http://clermontyping.iame-research.center/). The phylogenetic analysis was performed using Roary v.3.11.0 as already described (40). Pairwise SNP distances were calculated from core genome alignments generated by Roary using snp-dists (https://github.com/tseemann/snp-dists) (Table S2). Visualizing was performed using iTOL v6.5.2 (https://itol.embl.de/).

### MinION long-read sequencing

MinION long-read sequencing libraries were prepared according to the Oxford Nanopore Technologies using the native barcoding expansion kit (EXP-NBD104) and the ligation sequencing kit (SQK-LSK109). Sequencing was performed on a MinION sequencer using a SpotON Mk 1 R9 version flow cell (FLO-MIN106D). Assembly of both Illumina and Nanopore reads was performed using Unicycler. The assembled contigs were annotated using Bakta (Web version 1.7.0/DB: 5.0.0) (41).

### Characterization of plasmids

#### Molecular characterization

The genetic determinants carrying the ESBL/AmpC genes were detected by Southern blot on *I-Ceu*1- or S1-digested DNA Pulsed-Field Gel Electrophoresis (PFGE) as already described (42), using the DIG DNA Labeling and Detection Kit (Roche Diagnostics, Meylan, France) according to the manufacturer’s instructions.

#### *In silico* characterization

The PROKSEE server (https://proksee.ca/) was used to generate high-quality navigable maps of each circular plasmid described and the visualization of genetic environment of chromosomal genes (43). Circular comparison between the plasmids belonging to the same incompatibility group and the closest plasmids found in the NCBI database was carried out on the PROKSEE server using BLAST analysis (BLAST+ 2.12.0).

## Data Availability

The project was deposited in DDBJ/EMBL/GenBank under the BioProject accession number PRJNA976065.

## SUPPLEMENTAL MATERIAL

Fig. S1 spectrum.02220-23-s0001.tif Map of the sampling.

Table S1 spectrum.02220-23-s0002.xlsx List and characteristics of all *E. coli* and *E. marmotae* strains.

Table S2 spectrum.02220-23-s0003.xlsx SNP distances between all *E. coli* and *E. marmotae* isolates.

Table S3 spectrum.02220-23-s0004.xlsx Quality controls of all *E. coli* and *E. marmotae* data.

ASM does not own the copyrights to Supplemental Material that may be linked to, or accessed through, an article. The authors have granted ASM a non-exclusive, world-wide license to publish the Supplemental Material files. Please contact the corresponding author directly for reuse.
