# Supplementary figures and images for: Molecular characterization of highly prevalent Escherichia coli and Escherichia marmotae resistant to extended-spectrum cephalosporins in European starlings (Sturnus vulgaris) in Tunisia
Source: Microbiol Spectr. 2023 Sep 29;11(5):e02220-23. doi: 10.1128/spectrum.02220-23 (PMC10581222; doi:10.1128/spectrum.02220-23)

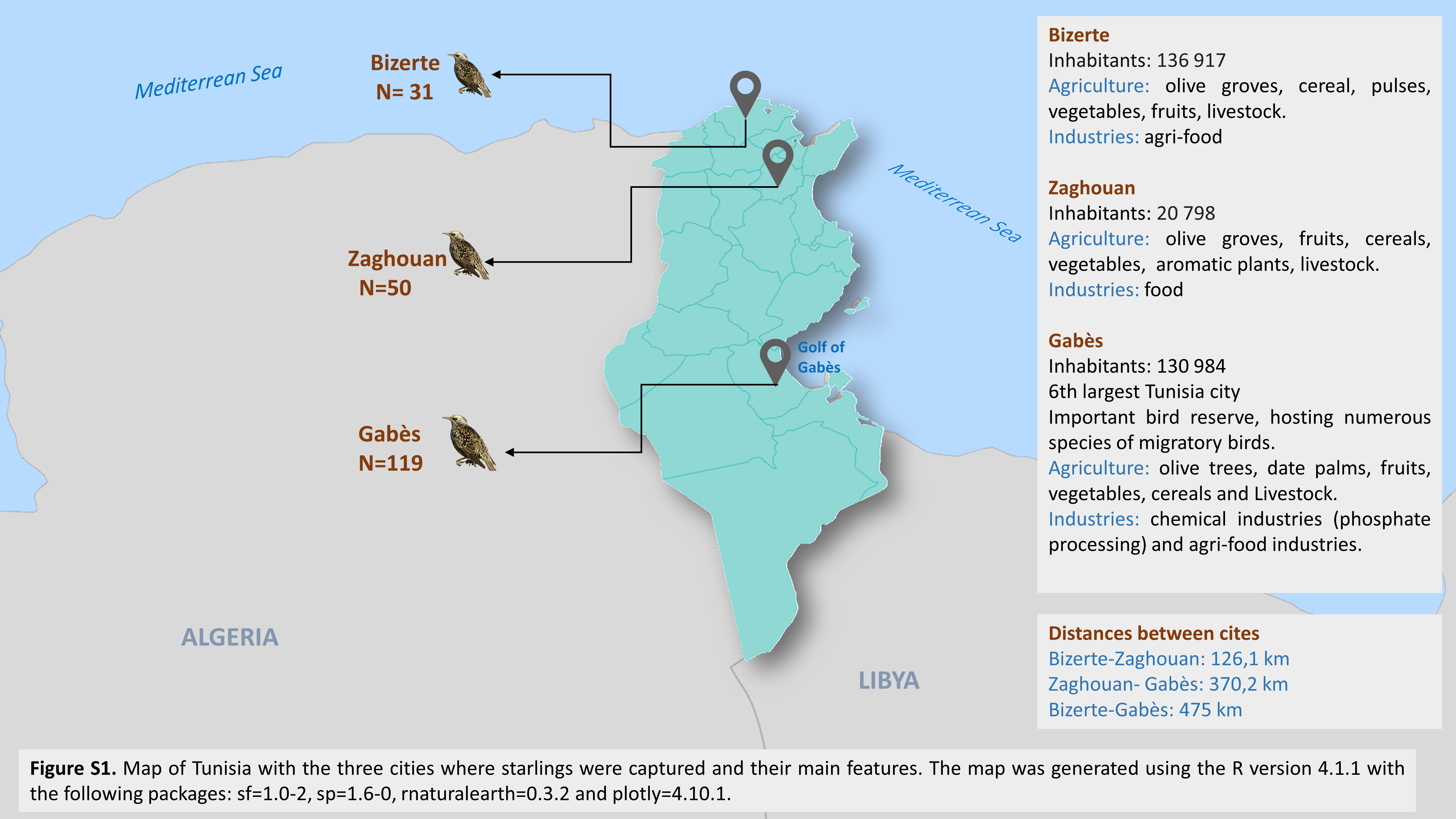

Supplement: Fig. S1 — Map of the sampling. [file spectrum.02220-23-s0001.tif]
